# A new species of *Rhopalopsole* (Plecoptera, Leuctridae) from Hainan Province, China

**DOI:** 10.3897/BDJ.12.e122999

**Published:** 2024-05-10

**Authors:** Xiao Yang, Yu-Zhou Du

**Affiliations:** 1 School of Plant Protection & Institute of Applied Entomology, Yangzhou University, Yangzhou 225009, China School of Plant Protection & Institute of Applied Entomology, Yangzhou University Yangzhou 225009 China

**Keywords:** Plecoptera, *Rhopalopsole*, new species, Hainan, China

## Abstract

**Background:**

Hainan Province is an island in the south of China and belongs to the Oriental Region. It has a unique geographical location and superior climatic conditions, providing a good living environment for Leuctridae insects. However, the species richness of the stonefly family Leuctridae in Hainan is low. Two species in total have been recorded, *Rhopalopsolebawanglinga* Li, Li & Yang, 2023 and *Rhopalopsolehainana* Li & Yang, 2010.

**New information:**

A new species of Leuctridae (Plecoptera) from Wuzhi Mountains, Hainan Province of south China, *Rhopalopsolewuzhishana* **sp. nov.** is described and illustrated. We summarised the Leuctridae in Hainan Province and provide supplemental description and colour plates of *Rhopalopsolehainana* Li & Yang, 2010.

## Introduction

*Rhopalopsole* Klapálek, 1912 is a species-rich genus in the family Leuctridae, with more than 80 valid species known from the Oriental and eastern Palaearctic Regions (Klapálek 1912, Okamoto 1922, Wu 1949, Jewett 1958, Kawai 1967, Kawai 1968, Kawai 1969, Wu 1973, Jewett 1975, Harper 1977, Zwick 1977, Harrison and Stark 2008, Sivec et al. 2008, Stark and Sivec 2008, DeWalt et al. 2024). Currently, more than 50 species of this genus have been recorded from China, with recent contributions made by Yang et al. (2004), Yang et al. (2006), Sivec et al. (2008), Yang et al. (2009), Li and Yang (2010), Li and Yang (2011), Qian and Du (2011), Li and Yang (2012), Qian and Du (2012a), Qian and Du (2012b), Qian and Du (2013), Chen and Du (2017), Li et al. (2017), Qian and Du (2017), Mo et al. (2018), Yang and Du (2021a), Yang and Du (2021b), Yang and Du (2021c), Yang and Du (2022), Yang et al. (2022)and Li et al. (2023).

Recently we examined a collection of Plecoptera from Hainan Provence. We describe and illustrate a new species of *Rhopalopsole* Klapálek, 1912, *Rhopalopsolewuzhishana*, sp. nov. In addition, we found *Rhopalopsolehainana* Li & Yang, 2010 in this collection and, because the original description is incomplete, we provide a supplementary description accompanied by high definition colour photographs.

## Materials and methods

Specimens were collected by hand and preserved in 75% ethanol. Morphological details were examined with a Leica MZAPO microscope. Colour illustrations were taken with a KEYENCE VHX-5000 digital dissection microscope. All specimens used in this study are deposited in the Insect Collection of Yangzhou University (ICYZU), Jiangsu Province, China. All the type materials are deposited ICYZU. The morphological terminology follows that of Sivec et al. (2008).

## Taxon treatments

### Rhopalopsole wuzhishana

Yang & Du sp. nov.

5BFD944B-C4F4-5459-A5A2-CAA65F63DDEB

CD84685A-7168-42AC-B890-DC6E6680003A

#### Materials

- **Type status:** Holotype. **Occurrence:** sex: male; lifeStage: adult; occurrenceID: E653CA80-E5DA-503B-BE44-779E9CA53E10; **Taxon:** scientificName: *Rhopalopsolewuzhishana*; order: Plecoptera; family: Leuctridae; genus: Rhopalopsol; specificEpithet: wuzhishana; **Location:** island: Hainan; country: China; stateProvince: Hainan; municipality: wuzhishan; locality: shuimanxiang; verbatimLatitude: 18.907855 N; verbatimLongitude: 109.679361 E; **Event:** eventDate: 2024-01-8T15:25-0800; **Record Level:** language: en
- **Type status:** Paratype. **Occurrence:** sex: 1 male; lifeStage: adult; occurrenceID: 4E138CE4-282E-5450-BCBC-78DA4A1937C6; **Taxon:** scientificName: *Rhopalopsolewuzhishana*; order: Plecoptera; family: Leuctridae; genus: Rhopalopsol; specificEpithet: wuzhishana; **Location:** island: Hainan; country: China; stateProvince: Hainan; municipality: wuzhishan; locality: shuimanxiang; verbatimLatitude: 18.907855 N; verbatimLongitude: 109.679361 E; **Event:** eventDate: 2024-01-8T15:25-0800; **Record Level:** language: en

#### Description

**Figure 1.**
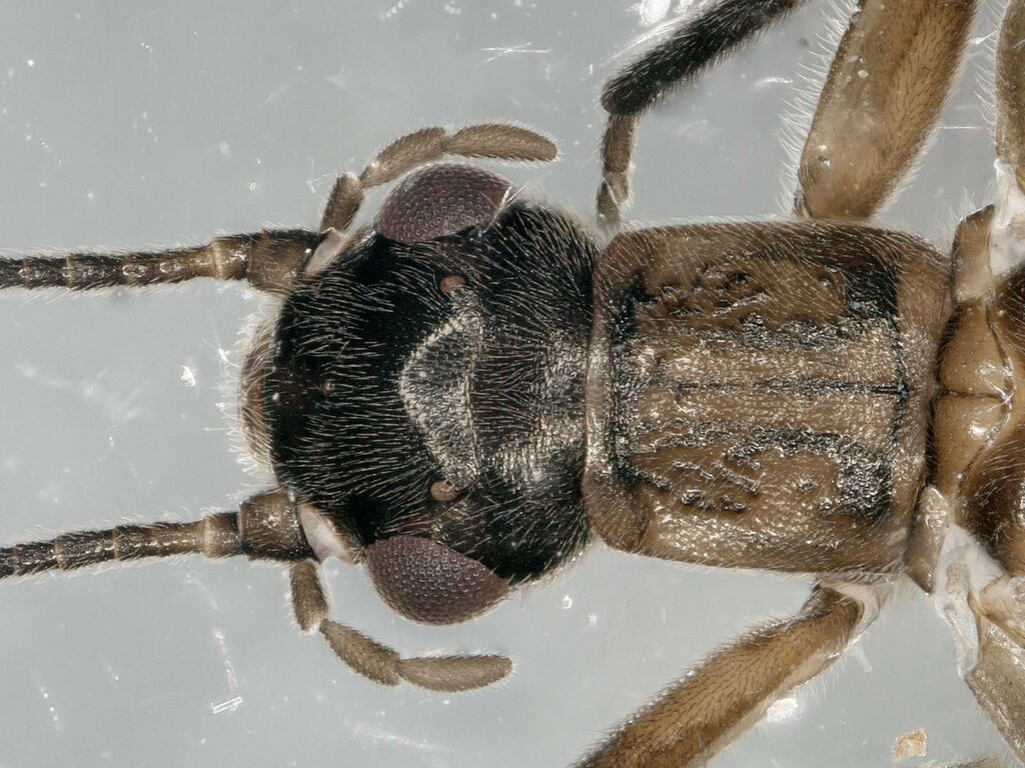
*Rhopalopsolewuzhishana*, sp. nov. Male head and pronotum, dorsal view.

Body length 5.5-5.6 mm. Fore-wings length 5.8-6.0 mm, hind-wings length 4.1-4.3 mm. Head dark brown, wider than pronotum; ocelli pale brown; antennae and palpi light brown. Pronotum brown, quadrate, all angles rounded. Legs dark brown. Wings hyaline and veins light brown (Fig. 1).

**Figure 2.**
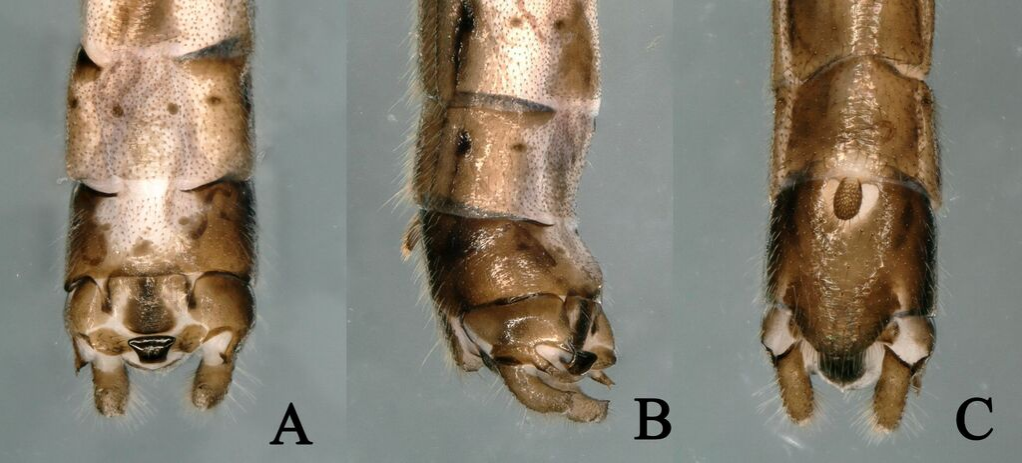
*Rhopalopsolewuzhishana*, sp. nov. **A** Male terminalia, dorsal view; **B** Male terminalia, lateral view; **C** Male terminalia, ventral view (before treatment with sodium hydroxide solution).

**Figure 3.**
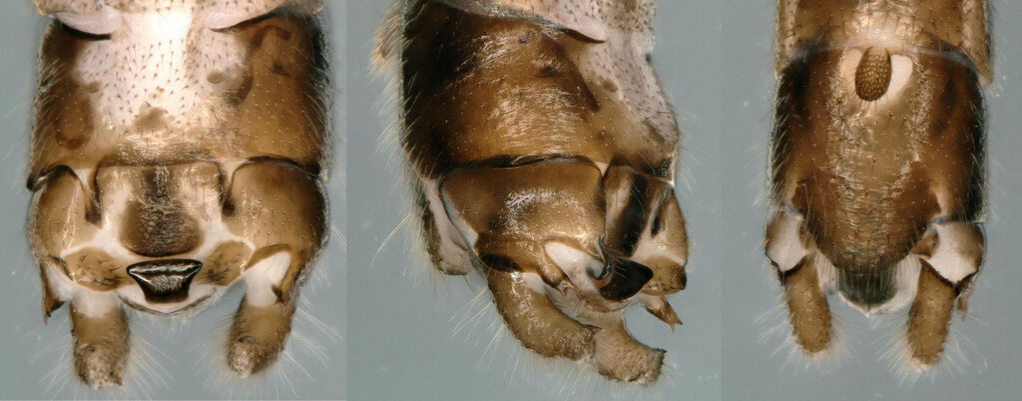
*Rhopalopsolewuzhishana*, sp. nov. **A** Male terminalia tergum 10, dorsal view; **B** Male terminalia tergum 10, lateral view; **C** Male terminalia tergum 10, ventral view (before treatment with sodium hydroxide solution).

Tergum 9 weakly sclerotised, with a wide, incised anterior margin that is more poorly sclerotised medially and continuing posteriorly to form a petal-like area with hairs. Sternum 9 basally long 1 x longer than width and with subcircular vesicle bearing dense hairs. Tergum 10 bearing a large central plate covered with a broad sensilla basiconica patch in the posterior half and somewhat less sclerotised in the anterior half. Transverse plates roughly oval with apically setae. Lateral projections of tergum 10 originating as a rounded base and extending upwards and backwards in a short plate, ending in a forked process, the lower point slightly larger. Epiproct oblate, size of the base to apex slowly increasing. Subanal lobes of medium size, flat and plate-like, expanding posteriorly, with well-defined ventral furrows. Cercus hairy and upcurved, with a small spine (Figs 2, 3).

#### Diagnosis

*Rhopalopsolewuzhishana* sp. nov. is a member of the *R.vietnamica* Sivec & Harper, 2008 west group as proposed by Sivec et al. (2008), with tergum 10 of the adult male possessing a central plate with lateral bars strongly sclerotised, lateral projections typically ending in a forked process, epiproct simple and thick and subanal lobes typical of this group. The new species is most similar to the *Rhopalopsolehainana* (Li and Yang 2010). The males share similarly-shaped subanal lobes and the shape of the lateral projections of tergum 10. In *R.wuzhishana*, the central plate of tergum 10 is larger. Tergum 9 is weakly sclerotised, with a wide, incised anterior margin that is more poorly sclerotised medially and continuing posteriorly to form a petal-like area with hairs. Sternum 9 basally teardrop-shaped and long 1 x longer than width and with subcircular vesicle bearing dense hairs. The epiproct is oblate, with a gradual increase in size from the base to the apex. However, in *R.hainana*, tergum 9 is weakly sclerotised, with a wide, incised anterior margin. In addition, the median anterior margin is more weakly sclerotised and extending posteriorly as a vase-like area with hairs orientated differently from those in lateral sections. The central plate of tergum 10 is smaller. Sternum 9 is basally rounded. Epiproct thick tip is narrow than *R.wuzhishana* sp.nov.

#### Etymology

This new species is named after the collection place.

#### Distribution

Hainan Province, China.

### Rhopalopsole hainana

Li & Yang, 2010

60E06808-B1B4-5EA2-89A2-A916DF645E75

#### Materials

- **Type status:** Other material. **Occurrence:** sex: 4 males; lifeStage: 4 adult; occurrenceID: EB13C7BE-0DE5-55A5-8D48-B34558F50388; **Taxon:** scientificName: *Rhopalopsolehainana*; order: plecoptera; family: Leuctridae; genus: Rhopalopsole; specificEpithet: *hainana*; **Location:** island: Hainan; country: China; stateProvince: Hainan; county: wuzhishan; verbatimLatitude: 18.702331N; verbatimLongitude: 109.679361E

#### Description

**Figure 4.**
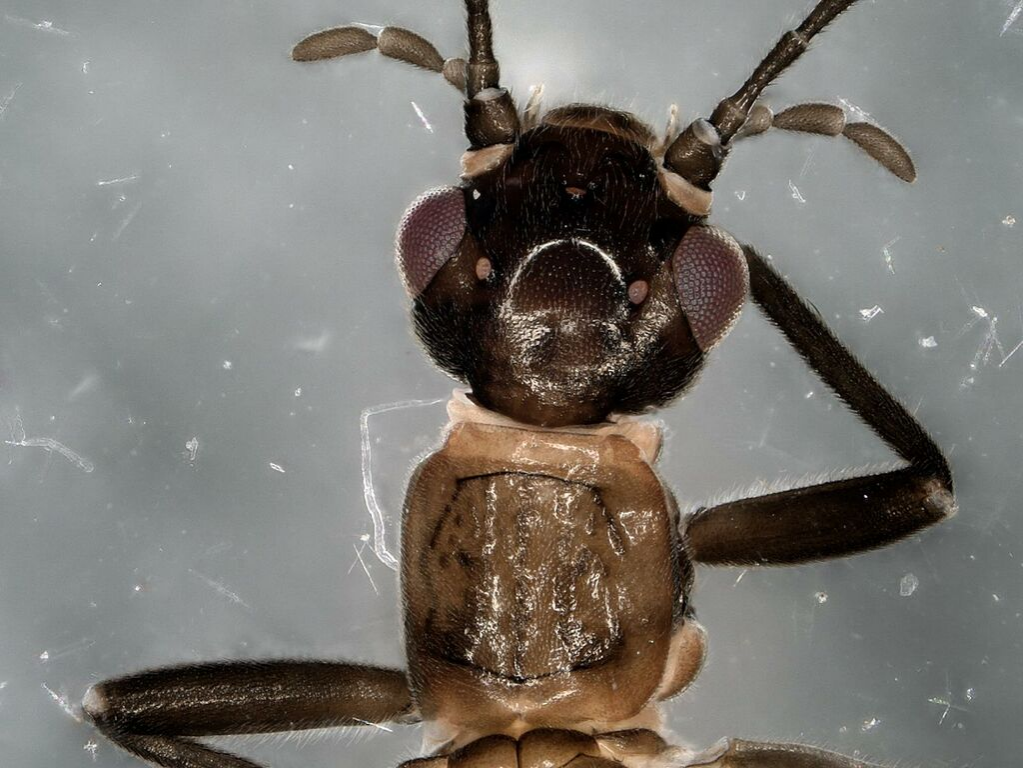
*Rhopalopsolehainana* Li & Yang, 2010. Male head and pronotum, dorsal view.

**Figure 5.**
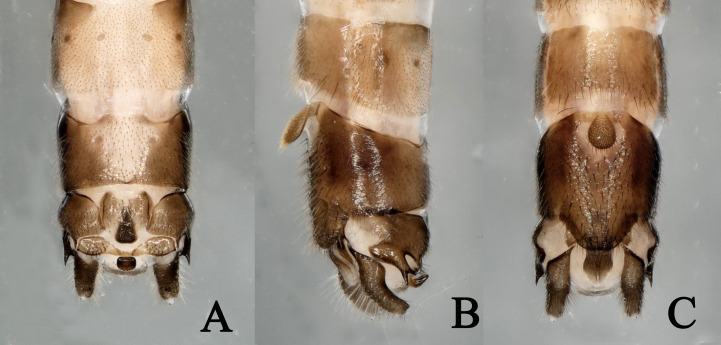
*Rhopalopsolehainana* Li & Yang, 2010. **A** Male terminalia, dorsal view; **B** Male terminalia, lateral view; **C** Male terminalia, ventral view.

This species was well described by Li and Yang (2010). Therefore, we only provide photographs in this paper (Figs 4, 5).

## Identification Keys

### Key to the males of *Rhopalopsole* Klapálek, 1912 from Hainan Province, China

**Table:** 

1	Epiproct with circularly incised anterior margin in dorsal view.	*R.bawanglinga* Li, Li & Yang
–	Epiproct without circularly incised anterior margin in dorsal view.	2
2	Tergum 9 poorly sclerotised medially and continuing posteriorly to form a vase-like area.	*R.hainana*, Li & Yang
–	Tergum 9 poorly sclerotised medially and continuing posteriorly to form a petal-like area.	*R.wuzhishana* Yang & Du, sp. nov.

## Discussion

Up to now, there are three species of Leuctridae in Hainan Province, *R.bawanglinga* Li, Li & Yang, *R.hainana* Li & Yang, 2010 and *R.wuzhishana* sp. nov. (Table 1). All three species belong to the *R.vietnamica* group, western assemblage (sensu Sivec et al. (2008)). We provided a key to retrieve *Rhopalopsole* in Hainan.

Although the collectors have collected from Hainan in a relatively comprehensive way, there are still many areas in the centre of Hainan Province that have not been investigated. In the future, we will collect specimens from uncollected areas.

## Supplementary Material

XML Treatment for Rhopalopsole wuzhishana

XML Treatment for Rhopalopsole hainana

## Figures and Tables

**Table 1.** Collection of Leuctridae in Hainan Province.

**Species**	**Latitude**	**Longitude**	**Comments**
*R.bawanglinga*	19.2504 N	109.034 E	holotype
	18.747 N	108.8492 E	paratype
*R.hainana*	18.707 N	108.8247 E	holotype
	19.0812 N	109.5197 E	
	19.2062 N	109.554 E	
	18.7253 N	109.869 E	
	18.702331 N	109.692115 E	
*R.wuzhishanensis*	18.907855 N	109.679361 E	holotype
